# Magnitude and Associated Factors of Podoconiosis and its Comorbidity With Tungiasis Among Residents in Southwest Ethiopia: A Community‐Based Cross‐Sectional Study

**DOI:** 10.1155/jotm/6163215

**Published:** 2026-03-08

**Authors:** Yared Nigusu, Sisay Teferi, Eshetu Chilo, Teshome Bekana, Kassahun Demelash Alemu, Samuel Ejeta Chibsa, Geleta Nenko Dube, Dereje Oljira Donacho

**Affiliations:** ^1^ Armauer Hansen Research Institute, Addis Ababa, Ethiopia, ahri.gov.et; ^2^ Department of Medical Laboratory Science, College of Health Sciences, Mattu University, Mattu, Ethiopia, meu.edu.et; ^3^ Department of Midwifery, College of Health Sciences, Mattu University, Mattu, Ethiopia, meu.edu.et; ^4^ School of Public Health, Institute of Health, Bule Hora University, Bule Hora, Ethiopia, bhu.edu.et; ^5^ Department of Health Informatics, College of Health Sciences, Mattu University, Mattu, Ethiopia, meu.edu.et

**Keywords:** associated factors, comorbidity, Ethiopia, magnitude, podoconiosis, tungiasis

## Abstract

**Background:**

Nonfilarial elephantiasis, also known as podoconiosis, is a completely preventable, neglected tropical disease characterized by prominent swelling of the lower extremities. The disease is common in sub‐Saharan Africa. However, its epidemiology varies from region to region. Its comorbidity with other diseases is also rarely studied in Ethiopia. A better understanding of podoconiosis and its comorbidity with tungiasis is crucial for the utmost consideration of the management and prevention strategies.

**Objective:**

The study aimed to assess the magnitude and associated factors of podoconiosis and its comorbidity with tungiasis among residents of southwest Ethiopia.

**Methods:**

A community‐based cross‐sectional study was conducted in selected districts of southwest Ethiopia from February to May 2023. After appropriate data collection and processing, the descriptive statistics were computed to determine the magnitude of podoconiosis and its comorbidity with tungiasis. A bivariate and multivariate logistic regression analysis was computed to identify the factors associated with podoconiosis in the frame of Hosmer–Lemeshow’s goodness of fit.

**Result:**

A total of 554 study participants were enrolled in the study. Podoconiosis was identified among 34 study participants, with a magnitude of 6.14% (95% CI: 4.21, 8.4%). Of the total podoconiosis‐affected study participants, about 11.8% (*n* = 4) were simultaneously infected with tungiasis. The disease was found to be associated with occupation, time not wearing shoes, usage of soap for leg/foot washing, and family history of leg swelling in the study participants.

**Conclusion:**

This study indicates a significant prevalence of podoconiosis and a notable comorbidity with tungiasis. The findings of this study highlight the need for targeted interventions on improved footwear practices and hygiene education in the study area. Moreover, potential genetic screening of the population in podoconiosis endemic areas can contribute to the early prevention of the disease, thus reducing its burden and alleviating the torment of the population.

## 1. Introduction

Neglected tropical diseases (NTDs) have gained significant global attention in recent years [1] since the reports of high incidence rates in the last 2 decades [2]. An estimated one billion people are infected with one or more NTDs, most of whom live in developing countries [3]. More than half of the 32 countries where nonfilarial elephantiasis (also called podoconiosis) has been endemic globally are found in Africa, and recent evidence showed that the prevalence as high as 8.08% in Cameroon and 7.45% in Ethiopia has been recorded [4].

East Africa is the region with the overwhelming NTD cases, and the prevalence of podoconiosis up to 7.2% was recorded in Uganda [3, 5]. Ethiopia is the third‐ranked country in Africa with the highest number of NTD cases, with podoconiosis, trachoma, and cutaneous leishmaniasis being the three most common [6]. About 1.5 million podoconiosis cases were reported in 2017, with more than 172,000 disability‐adjusted life years (DALYs) costing about US$213.2 million in total economic burden annually in the country [7]. Despite the burden and jeopardizing situation of podoconiosis in Ethiopia [7–10], the effort and concerns given to alleviate the burden of podoconiosis are not as high as its public health impact.

The impact of podoconiosis is not limited to leg swelling and economic burden only, but also extends to the cultural and social stigma [11, 12]. Individuals with podoconiosis face difficulties in attending education and finding employment, and are even prohibited from marrying unaffected people [13] because of a belief that suggests podoconiosis is hereditary [14]. Therefore, the disease has been advancing from a neglected disease to a considerable public health problem as a result of its double burden of economic (subsequent decline in family income) [15] and social (stigma) [13] impacts on both patients and their families.

Besides, several factors have been driving podoconiosis to be a profound public health problem in communities with low socioeconomic status [9]. Evidence suggests that low shoe‐wearing habits, low level of education, age at first shoe‐wearing, low feet washing practice, low wealth index, minimal number of shoes owned, low utilization of soap during feet washing, and family history with a similar disease are some of the determinants of podoconiosis [16–18].

However, some of the determinants of podoconiosis are also associated with another NTD, tungiasis, a disease characterized by the infestation of a sand flea (*Tunga penetrans*) under the skin and predominantly affecting populations of low‐ and middle‐income countries [19, 20], where low shoe‐wearing habits, low level of education, and low socioeconomic status are also considered determinants of tungiasis [21–23].

In sub‐Saharan Africa (SSA), about one‐third of the population is infected with tungiasis. Evidence from eastern Africa reveals that six of 10 individuals were affected by tungiasis, with the prevalence of tungiasis in humans as high as 62.8% in northeastern Uganda [24].

Similarly, a significant population was infected with tungiasis in Ethiopia as well, and the prevalence of around 58% was recorded [23]. Hence, in the presence of overlapping significant factors associated with both podoconiosis and tungiasis, there could be a potential comorbidity of the diseases, which further intensifies the burden of podoconiosis. Data from Uganda indicate the significant comorbid cases of podoconiosis and tungiasis, revealing that about 16.6% of study participants affected by podoconiosis were simultaneously infected by tungiasis [5]. However, there are no clear data in Ethiopia that witness the comorbidity between both diseases.

So far, the available evidence suggests that significant attention should be directed toward podoconiosis. As a result, certain concerned bodies have been working on controlling and preventing podoconiosis in Ethiopia over the last decades. However, the burden of the disease has remained significant in recent years, too, causing considerable morbidity and impaired health‐related quality of life [25]. Hence, it is important to identify the factors contributing to the steady burden of podoconiosis in the community. Moreover, there is no direct evidence of comorbidity between podoconiosis and tungiasis, though both diseases share some risk factors. A better understanding of podoconiosis and its comorbidity with tungiasis contributes to the utmost consideration of the management and prevention strategies by the concerned bodies. Therefore, this study aimed to assess the magnitude and associated factors of podoconiosis and comorbidity with tungiasis in southwest Ethiopia.

## 2. Materials and Methods

### 2.1. Study Setting and Period

A study was conducted in selected districts of the Ilu Ababor zone, Oromia region, southwest Ethiopia, from February to May 2023. The Oromia region is characterized by diverse landscapes, including highlands, valleys, and lowland areas ranging from 1000 to 4600 m above sea level. The region has several highland areas with fertile soils and diverse crop production. The Ilu Ababor zone is one of the zones in the Oromia region, located 538 km away from the capital city of the country, Addis Ababa, at an elevation of 1605 m above sea level. According to the Ethiopian Central Statistical Agency population projection of 2023, the zone has a total population of about 1.9 million residing in 13 districts, of whom more than 85% are rural inhabitants. Due to the region’s high elevation, the highland areas have significant rainfall and erosion, which exposes the soil particles to prolonged contact with the skin. The clay‐rich soils in these regions often contain irritant minerals, which contribute to the development of podoconiosis.

### 2.2. Study Design and Population

A community‐based cross‐sectional study was carried out among residents in selected districts of the Ilu Ababor zone, southwest Ethiopia.

### 2.3. Sample Size Determination

For the assessment of the magnitude of podoconiosis, the minimum representing sample size was calculated using a single population proportion formula, considering a 5.7% previous prevalence of podoconiosis in a country [9], a 95% confidence interval, a 3% margin of error, design effect of 1.5%, and 5% nonresponse rate. The minimum sample size was computed to be 344.

The sample size for the associated factors was determined by Epi Info Version 7 statistical software. Results from similar studies [17, 26, 27] were used to approximate the sample size with different potential associated factors of podoconiosis. Accordingly, we have used P1: proportion of outcome in unexposed, P2: proportion of outcome in exposed, adjusted odds ratio (AOR), ratio of unexposed to exposed, 95% confidence interval, and power of assumption of 80%. Therefore, the sample size used for the study was the highest from the sample size calculated for the prevalence and associated factors, which was 554 (Table 1).

**TABLE 1:** Epi Info sample size calculation for associated factors of podoconiosis in southwest Ethiopia, 2023.

Variables	Ratio	*P*_1_	*P*_2_	AOR	Final sample size with 5% NRR
Family history of leg swelling	5.38	4.1	16.7	4.69	353
Regular barefoot walking	1.2	4.94	15.15	3.44	321
Feet washing habits	1.37	1.96	7.48	3.87	554

*Notes:* *P*_1_, proportion of outcome in unexposed; *P*_2_, proportion of outcome in exposed.

Abbreviations: AOR = adjusted odds ratio, NRR = nonresponse rate.

### 2.4. Sampling Technique

A multistage sampling technique was used, and 5 of 13 districts, Alle, Yayo, Bure, Bacho, and Hurumu, with altitudes of 2084 m, 1732 m, 1806 m, 1963 m, and 1739 m, respectively, were selected randomly. Then, three smallest administrative units (kebeles) were randomly selected from each district, and a total of 15 kebeles were involved in the study. A lottery method was used for the selection of study participants from the randomly selected and proportionally allocated households of each kebele.

### 2.5. Eligibility Criteria

#### 2.5.1. Inclusion Criteria

All residents who have been living for more than 6 months in the study area and are above 18 years old during the study period were eligible for the study.

#### 2.5.2. Exclusion Criteria

Individuals with clinical signs and symptoms of lymphatic filariasis (evidence supported by the filarial test strip), diabetic neuropathy, and edema of known origin were excluded.

### 2.6. Data Collection Procedure

#### 2.6.1. Sociodemographic Data

A pretested structured questionnaire was used to collect sociodemographic characteristics and associated factors of podoconiosis through face‐to‐face interviews conducted by 15 trained data collectors who had a minimum of diploma in nursing.

#### 2.6.2. Identification of Individuals with Podoconiosis

An individual with podoconiosis was diagnosed by trained nurses. A participant was considered positive for podoconiosis in case there had been a history of burning sensation in the feet when the swelling started and visible swelling that started at the feet and progressed upward, with at least stage one of the five clinical stages of podoconiosis [28].

#### 2.6.3. Identification of Individuals for Comorbidity with Tungiasis

Comorbidity with tungiasis was identified when a person with podoconiosis undergoes physical examination of jigger infestation characteristics using the Fortaleza classification: from a dark and scraping spot in the skin to distinct craters, such as sores or supportive lesions in the natural history of illness [29].

### 2.7. Data Processing and Analysis

EpiData Software Version 3.1 and IBM SPSS Statistics for Windows, Version 22 (IBM Corp., Armonk, NY, USA) were used for data entry and analysis, respectively. Descriptive statistics were computed for the sociodemographic, behavioral, and clinical characteristics of study participants and the determination of the magnitude of podoconiosis. Bivariate logistic regression analysis was conducted, and variables with a *p*‐value ≤ 0.25 were selected as candidate variables for multivariable logistic regressions to identify the possible factors associated with podoconiosis. A *p*‐value of ≤ 0.05 was used to pinpoint a statistically significant association.

## 3. Results

### 3.1. Sociodemographic Characteristics and Housing Conditions of the Study Participants

A total of 554 study participants were enrolled, of whom 42.2% were males and 57.8% were females. The mean age of the participants was 41.45 + 11.34. About 35.0% of the study participants were those with no formal education. The majority, 38.6% of the study participants, were farmers (Table 2).

**TABLE 2:** Sociodemographic characteristics and housing condition of study participants in selected districts of the Ilu Ababor zone, southwest Ethiopia, 2023 (*N* = 554).

Variables	Categories	*N* (%)
Age	18–24	83 (9.6)
	25–34	138 (17.1)
	35–44	176 (28.2)
	45–54	95 (23.1)
	≥ 55	62 (22.0)

Sex	Male	234 (42.2)
	Female	320 (57.8)

Level of education	No formal education	194 (35.0)
	1–8	106 (19.2)
	9–12	67 (12.1)
	Diploma	122 (22.0)
	First‐degree and above	65 (11.7)

Occupation	Agriculture workers	214 (38.6)
	Government employees	211 (38.0)
	Private sector workers	102 (18.5)
	Others^∗∗^	27 (4.9)

The main source of water	Piped water	432 (78)
	Protected spring	63 (11.4)
	Unprotected spring	35 (6.2)
	Surface water	24 (4.4)

Distance from the house to the water source	In the living compound	93 (16.8)
	< 1 km	175 (67.7)
	≥ 1 km	286 (15.5)

The living room floor	Earth and mud	437 (78.9)
	Cement	113 (20.4)
	Ceramic tiles	4 (0.7)

The main living house roof	Iron sheet	488 (88.1)
	Grass/leaf	66 (11.9)

The main living room wall	Wood and mud	433 (78.2)
	Wood and cement	117 (21.1)
	Stone with cement	4 (0.7)

*Note:* N, number. agriculture workers are those participating in either land farming and/or livestock farming; private sector workers are those employed in private institutions and/or companies. ^∗∗^unemployed, street vendors, daily laborers, and self‐employed.

### 3.2. Behavioral and Other Characteristics of the Study Participants

Of the total study participants, nearly two‐thirds do not wear shoes while they are either at home or at the workplace. Most of the study participants wash their legs/feet only once per day (*n* = 288, 52.9%), and nearly half of the study participants did not use soap for leg/foot washing (*n* = 264, 47.7%). Over half of the study participants did not wear shoes during their childhood (less than 14 years old), while nearly one‐third of the study participants had a family history of podoconiosis (*n* = 161, 29.1%) (Table 3).

**TABLE 3:** Behavioral and other characteristics of the study participants in selected districts of the Ilu Ababor zone, southwest Ethiopia, 2023 (*N* = 554).

Variables	Categories	*N* (%)
Time of not wearing shoes	At workplace	147 (26.5)
	At home	206 (37.2)
	Wear always	201 (36.3)

Leg/feet washing frequency per day	Once	288 (52.9)
	Twice	195 (35.2)
	Three times	71 (12.8)

Use of soap for leg/foot washing	Yes	290 (52.3)
	No	264 (47.7)

The age at which shoe‐wearing started	< 5	119 (21.5)
	6–14	162 (29.2)
	15–24	187 (33.8)
	> 25	86 (15.5)
Family history of podoconiosis	Yes	161 (29.1)
	No	393 (70.9)

*Note:* N, number.

### 3.3. Magnitude of Podoconiosis

The prevalence of podoconiosis was 6.14% (*n* = 34) (95% CI: 4.21, 8.4%). The highest prevalence of podoconiosis was observed among age groups > 55 years old (*n* = 14, 41.2%) and female study participants (*n* = 21, 61.7%), with a female‐to‐male ratio of 1.6:1.

### 3.4. Clinical Characteristics of the Study Participants with Podoconiosis

Nearly two‐thirds of the study participants with podoconiosis were first observed with leg swelling in the age range of 25–34 (*n* = 21, 63.6%), with the average age of first observed leg swelling at 32.5 years old. More than 90% of the individuals with podoconiosis (*n* = 31) had a fever during the active phases, and about two‐thirds had tenderness. Mossy lesions and open wounds were seen in 38.2% (*n* = 13) and 23.5% (*n* = 8) of patients, respectively. About two‐thirds of the study participants with podoconiosis were complaining that the problem worsened during the chagino season (the late rainy season or early dry season or the transitional period between the heavy rainy season of June to August and the drier season of October to February, characterized by decreasing rainfall, sunny days, and cooler temperatures). The majority of the study participants with podoconiosis remained housebound/admitted during active phases of the disease (*n* = 28, 82.4%) (Table 4).

**TABLE 4:** Characteristics of the study participants with podoconiosis in selected districts of the Ilu Ababor zone, southwest Ethiopia, 2023 (*N* = 34).

Variables	Categories	*N* (%)
The age at which leg swelling was first considered	< 14	—
	15–24	3 (8.8)
	25–34	19 (55.9)
	35–44	7 (20.6)
	> 45	5 (14.7)

Major signs and symptoms	Swollen lymph nodes	34 (100)
	Oozing	30 (88.2)
	Tenderness	24 (70.1)
	Hot on touch	23 (67.6)
	Fever	31 (91.2)

The time of the year when the problem worsens	Rainy season	7 (20.6)
	The hot and dry season	4 (11.8)
	During chagino season	23 (67.6)

Mossy lesion	Yes	13 (38.2)
	No	21 (61.8)
Open wounds	Yes	8 (23.5)
	No	26 (76.5)

Presence of admission/being housebound during the active phase	Yes	28 (82.4)
	No	6 (17.6)

*Note:* N, number.

### 3.5. Factors associated with Podoconiosis

The bivariate logistic regression analysis revealed that gender, occupation, level of education, distance from the house to the main source of water, living house floor, time not wearing shoes, usage of soap for leg/foot washing at least once per day, and family history of leg swelling were the candidate variables with *p*‐values of ≤ 0.25. In multivariable logistic regression analysis, occupation, time not wearing shoes, usage of soap for leg/foot washing, and family history of leg swelling were significantly associated with nonfilarial elephantiasis.

According to our study, agriculture workers were 3.2 times more likely to be affected by nonfilarial elephantiasis than government employees (AOR: 3.2, 95%CI: [1.25, 7.64], *p* = 0.003). The odds of developing nonfilarial elephantiasis among the study participants who did not wear shoes when they were at the workplace were 5.23 times higher than the study participants who always wore shoes (AOR: 5.23, 95%CI: [1.83, 15.4], *p* = 0.001). However, the study participants who did not use soap for leg/foot washing at least once per day had 4.3 times higher odds of being affected by nonfilarial elephantiasis than those who used soap at least once per day (AOR: 4.3, 95%CI: [1.88, 9.72], *p* = 0.02). Likewise, the study participants who had a family history of podoconiosis were 2.47 times more likely to be affected by podoconiosis than the study participants with no family history of nonfilarial elephantiasis (AOR: 2.47, 95%CI: [1.28, 5.51], *p* = 0.033) (Table 5).

**TABLE 5:** Factors associated with podoconiosis in selected districts of southwest Ethiopia, 2023.

Variables	Categories	Pos. *N* (%)	Neg. *N* (%)	COR (95%CI)	AOR (95%CI)
Sex	Male	13 (5.5)	221 (94.4)	1	1
	Female	21 (6.6)	299 (93.4)	1.2 (0.59, 2.44)	2.2 (1.01, 5.23)

Occupation	Gov’t employees	7 (3.3)	204 (96.7)	1	1
	Agriculture workers	24 (11.2)	190 (88.8)	3.68 (1.55, 8.74)	3.2 (1.25, 7.64)^∗^
	Private workers	3 (2.9)	99 (97.1)	0.88 (0.22, 3.48)	0.82 (0.18, 3.11)
	Others^∗∗^	—	27 (100)	0.49 (0.03, 8.92)	0.33 (0.01, 7.98)

Level of education	No formal education	15 (7.7)	179 (92.3)	11.3 (0.68, 191.8)	9.14 (0.3, 182.1)
	1–8	9 (8.5)	97 (91.5)	12.8 (0.73, 223.1)	9.62 (0.5, 194.3)
	9–12	7 (10.4)	60 (89.6)	16.2 (0.9, 290.4)	12.6 (0.6, 222.7)
	Diploma	3 (8.8)	119 (91.2)	3.83 (0.19, 75.4)	2.14 (0.06, 63.4)
	First degree/above	—	65 (100)	1	1

Distance from the house to the main water source	In the compound	2 (2.2)	91 (97.8)	1	1
	≤ 1 km	10 (5.7)	165 (94.3)	2.75 (0.59, 12.85)	1.67 (0.24, 9.68)
	> 1 km	22 (64.7)	264 (35.3)	3.79 (0.87, 16.44)	2.41 (0.47, 11.6)

Living house floor	Earth or mud	31 (7.1)	406 (92.9)	0.69 (0.03, 13.24)	0.32 (0.02, 9.62)
	Cement	3 (2.7)	110 (97.3)	0.28 (0.01, 6.83)	0.26 (0.01, 6.55)
	Ceramic tiles	—	4 (100)	1	1.

Time not wearing shoes	At workplace	24 (17.7)	123 (82.9)	**7.64 (2.84, 20.6)**	**5.23 (1.83, 15.4)** ^**∗**^
	At home	5 (2.4)	201 (97.6)	0.98 (0.28, 3.42)	0.62 (0.13, 2.87)
	Wear always	5 (1.5)	196 (98.5)	1	1

Usage of soap for leg/foot washing	At least once per day	6 (2.1)	284 (97.9)	1	1
	Not at all	28 (10.6)	236 (89.4)	**5.61 (2.29, 13.79)**	**4.3 (1.88, 9.72)∗**

Family history of leg swelling	Yes	19 (11.8)	142 (88.2)	**3.37 (1.67, 6.81)**	**2.47 (1.28, 5.51)∗**
	No	15 (3.8)	378 (96.2)	1	1

*Note:* N, number; 1, reference category; numbers in bold font represent variables significantly associated with nonfilarial elephantiasis in multivariate logistic regression.

Abbreviations: AOR = adjusted odds ratio, COR = crude odds ratio.

^∗^ *p* < 0.05, ^∗∗^unemployed, street vendors, daily laborers, and self‐employed.

### 3.6. Comorbidity of Podoconiosis with Tungiasis

Of 34 study participants who had been affected by podoconiosis, 4 (11.8%) were simultaneously found to be infected by *Tunga penetrans* and thus are positive for tungiasis. All tungiasis‐positive study participants belonged to the age group 25–34. Three of four podoconiosis–tungiasis comorbid cases were found among female participants and those who did not have formal education (Table 6).

**TABLE 6:** Comorbidity of podoconiosis with tungiasis in selected districts of the Ilu Ababor zone, southwest Ethiopia, 2023 (*N* = 4).

Variables	Categories	Podoconiosis cases *N* (%)	Comorbidity *N* (%)
Sex	Male	13 (38.2)	1 (25)
	Female	21 (61.8)	3 (75)

Age group	18–24	—	—
	25–34	3 (8.8)	4 (100)
	35–44	8 (23.5)	—
	45–54	9 (26.5)	—
	> 55	14 (44.2)	—

Level of education	No formal education	15	3 (75)
	1–8	9	1 (25)
	9–12	7	—
	Diploma	3	—

*Note: N*, number.

## 4. Discussion

Chronic exposure to irritant particles from red clay soil is considered a possible cause of nonfilarial elephantiasis, which is the second most common cause of tropical lymphedema after filarial elephantiasis [23]. With regard to the insufficient consideration given to nonfilarial elephantiasis and related NTDs in Ethiopia, this study aimed to assess the magnitude and associated factors of podoconiosis and its comorbidity with tungiasis.

This study elucidated that the prevalence of nonfilarial elephantiasis in southwest Ethiopia was 6.14%, which is in line with the survey conducted in the southern part of the country, where the prevalence was 6.2% [17]. However, the finding of this study was higher as compared to the recent studies conducted in different parts of Africa, where the prevalence of 4.52% in Uganda [30], 3.1% in Kenya [31], and 3.87% in Tanzania [30] has been recorded. Similarly, this finding was higher than the findings of domestic studies conducted in southern Ethiopia (5.4%) [32] and western Ethiopia (2.8%) [33], and (3.05%) [28]. Some shreds of evidence also showed that there was a higher prevalence of podoconiosis in Uganda (7.2%) and in some regions of Ethiopia than the findings of this study [5, 34].

The inconsistency between the studies can be due to the variations in the occupational status of the study participants, which might/might not allow shoe‐wearing, as seen in our study, where most of the study participants were farmers. Additionally, the high prevalence of nonfilarial elephantiasis in the past years in Ethiopia compared to other African countries might contribute to the high prevalence recorded by this study because of the evidence that podoconiosis has a significant association with a family history of leg swelling [35]. Moreover, the variations in socioeconomic status, level of awareness of the community, soil types, differences in the study period, variation in initiation period and duration of interventional programs, and effectiveness of the interventional programs can also be the reasons.

This study revealed that the odds of agriculture workers being affected by nonfilarial elephantiasis were 3.2 times higher than that of government employees (AOR: 3.2, 95% CI: 1.25, 7.64). This finding is consistent with the studies conducted in different parts of the world [36, 37]. The possible reason is the minimal shoe‐wearing habit of the subsistence farmers due to the nature of their occupation, so that they have frequent and long‐term barefoot exposure to irritant soil particles, which might stimulate a provocative reaction in the lymphatic system, in turn causing thickening and obstruction of the lymphatic system.

Similarly, the study participants who did not wear shoes at the workplace had higher odds of contracting nonfilarial elephantiasis as compared to those who always wore shoes (AOR: 5.23, 95% CI: 1.83, 15.4). This finding agrees with the findings of other studies conducted in different parts of the country [17, 26, 27, 38]. The direct contact between skin and mineral particles might enhance the entrance of the particles through the skin and cause an inflammatory response of leg swelling. Evidence also showed that wearing shoes consistently from the early stage is effective in preventing nonfilarial elephantiasis [39].

However, individuals who do not use soap at least once per day for leg/foot washing are more likely to be affected by nonfilarial elephantiasis than those using soap at least once per day (AOR: 4.3, 95% CI:1.88, 9.72), which is in line with the finding of the other studies [16, 17, 32]. This can be due to the reason that consistent foot washing using soap helps to remove soil particles that trigger the inflammatory response and minimize the risk of contracting nonfilarial elephantiasis.

Moreover, this study has revealed that the study participants who have a family history of leg swelling are more likely to be affected by nonfilarial elephantiasis than their counterparts (AOR: 2.47, 95% CI: 1.28, 5.51). This finding is consistent with the findings of other studies conducted in Rwanda [40] and other parts of Ethiopia [17, 18]. This might be because of the scientific reason that there are inherited specific human leukocyte antigen class II (HLA class II) alleles that have been associated with podoconiosis, suggesting an immune‐mediated component and involvement in immune response regulation, thus increasing the podoconiosis risk within the family members [41]. Additionally, there might be the possibility of sharing similar socioeconomic status, geographical area, occupation, access to safe water, and cultural practices of walking barefoot within the family. Therefore, in the presence of inherited genetic susceptibility and favorable conditions for chronic exposure to irritant soil particles possibly shared among family members, there might be a risk of contracting podoconiosis.

However, this study also identified the possibility of the study participants with podoconiosis being infected by tungiasis, with the magnitude of comorbidity being 11.8%. This finding aligns with the study conducted in Uganda, where infection with *Tunga penetrans* is associated with nonfilarial elephantiasis with 16.6% comorbidity [5]. The possible reason for the comorbidity might be due to the overlapping of factors associated with podoconiosis and tungiasis. Foot hygiene, exposure to soil, occupation, socioeconomic characteristics, and shoe‐wearing habits are significantly associated with tungiasis [42–44] and podoconiosis [18, 27, 38]. However, the co‐occurrence (sharing of the same geographical regions, tropical region) might also be the reason for comorbidity [4, 45]. Furthermore, the skin damage caused by podoconiosis might enhance the infection with *Tunga penetrans*.

## 5. Conclusion

This study reported a noticeable magnitude of podoconiosis in southwest Ethiopia. The finding implies that the magnitude of nonfilarial elephantiasis in southwest Ethiopia is remarkably steady, as seen in the previous studies. Occupation, time not wearing shoes, usage of soap for leg/feet washing, and family history of leg swelling were factors associated with podoconiosis. The family history of leg swelling and possible immune response toward irritant soil particles, which can be common among family members, can create a smooth path for contracting nonfilarial elephantiasis. There was the possibility of infestation of tungiasis among the study participants with podoconiosis. However, most of the associated factors were those that can be easily improved by awareness creation on shoe‐wearing and leg/foot hygiene. Additionally, the socioeconomic support of the community helps reduce direct contact with soil particles. Potential genetic screening of the population where podoconiosis is endemic can identify individuals at risk and contribute to the early prevention of the disease. In case the genetic screening seems unlikely, we recommend the regular community‐based surveillance and family‐based intervention focusing on sanitary education and provision of footwear to the family members with a history of leg swelling. Moreover, sustainable interventions on shoe‐wearing and leg/foot hygiene of the entire population of susceptible areas are vital in dwarfing the torment of the community from nonfilarial elephantiasis.

## Author Contributions

All authors have a priceless contribution to the success of this study. Yared Nigusu: Formal analysis, methodology, and writing–review and editing. Sisay Teferi: Supervision and writing–review and editing. Eshetu Chilo: Writing–original draft, investigation, and methodology. TB: Writing–original draft, conceptualization, and supervision. Kassahun Demelash Alemu: Writing–original draft and supervision. Samuel Ejeta Chibsa: Writing–review and editing, formal analysis, and investigation. Geleta Nenko Dube: Writing–review and editing, formal analysis, and methodology. Dereje Oljira Donacho: Writing–review and editing, conceptualization, and data curation.

## Funding

No funding was received for this research.

## Ethics Statement

Ethical clearance was obtained from the Mattu University’s College of Health Sciences Ethical Review Committee with a reference number: RPG/295. The study protocol was conducted according to the Declaration of Helsinki. The study participants were provided with overviews of the study, the harms, and the benefits of participation. Then, formal consent was taken from the randomly selected study participants regarding the data collection. The positive cases of podoconiosis and tungiasis were linked to the nearest health facilities for appropriate management of the disease.

## Conflicts of Interest

The authors declare no conflicts of interest.

## Data Availability

The data that support the findings of this study are available from the corresponding author upon reasonable request.
